# Pycnodysostosis: Report of Two Novel CTSK Variants in a Child

**DOI:** 10.3390/clinpract16060115

**Published:** 2026-06-16

**Authors:** Daniela Trotta, Rossella Ferrante, Michele Sallese, Marianna Viele, Claudia Rossi, Vincenzo Scorrano, Sara Savelli, Milena Catenaro, Vincenzo De Laurenzi, Maurizio Aricò

**Affiliations:** 1 Department of Pediatrics, S. Spirito Hospital, Azienda Sanitaria Pescara, 65124 Pescara, Italy; daniela.trotta@asl.pe.it (D.T.); milena.catenaro@asl.pe.it (M.C.); 2 Center for Advanced Studies and Technology (CAST), “G. d’Annunzio” University of Chieti-Pescara, 66100 Chieti, Italy; rossella.ferrante@unich.it (R.F.); marianna.viele@phd.unich.it (M.V.); claudia.rossi@unich.it (C.R.); vincenzo.scorrano@unich.it (V.S.); vincenzo.delaurenzi@unich.it (V.D.L.); 3 Department of Innovative Technologies in Medicine and Dentistry, “G. d’Annunzio” University of Chieti-Pescara, 66100 Chieti, Italy; 4 Department of Science, “G. d’Annunzio” University of Chieti-Pescara, 66100 Chieti, Italy; 5 Imaging Department, Bambino Gesù Children’s Hospital IRCCS, 00165 Rome, Italy; sara.savelli@opbg.net

**Keywords:** pycnodysostosis, CTSK, cathepsin K, skeletal dysplasia, novel variants, osteosclerosis, osteoclast function

## Abstract

**Background:** Pycnodysostosis is a rare autosomal recessive skeletal disorder caused by biallelic pathogenic variants in *CTSK*, which encodes cathepsin K, a lysosomal cysteine protease required for osteoclast-mediated degradation of bone matrix. **Case Report:** We describe a girl with short stature, skeletal deformities, osteosclerosis, craniofacial features, clavicular dysplasia, and radiological evidence of fractures. Clinical exome sequencing identified two heterozygous *CTSK* variants, c.85T > C (p.Trp29Arg) and c.679A>T (p.Ile227Phe), both currently classified as variants of uncertain significance. Segregation analysis showed that the variants were inherited in trans. Computational modeling and in silico prediction tools supported a possible deleterious effect on cathepsin K structure or function. Serum cathepsin K was higher in the patient than in two age-matched controls; this result is reported as an exploratory observation only. Increased serum cathepsin K may reflect altered expression, secretion, clearance, or accumulation of dysfunctional protein, but cannot be interpreted as proof of compensatory upregulation. **Conclusions:** The patient’s clinical and radiographic features, the biallelic trans configuration of the *CTSK* variants, their rarity in population databases, and computational predictions support p.Trp29Arg and p.Ile227Phe as strong candidate disease-associated variants. Functional studies are required to confirm their effect on cathepsin K expression, maturation, and enzymatic activity.

## 1. Introduction

Pycnodysostosis (MIM #265800) is an extremely rare autosomal recessive skeletal disorder, with an estimated prevalence of approximately 1–1.7 cases per million births [1,2]. It is caused by homozygous or compound-heterozygous pathogenic variants in CTSK [3,4], which encodes cathepsin K, a lysosomal cysteine protease secreted by osteoclasts and required for degradation of bone matrix proteins.

The disease is characterized primarily by skeletal abnormalities. Typical features include delayed closure of cranial fontanelles, wormian bones, midface hypoplasia, dental anomalies, generalized osteosclerosis, and acro-osteolysis of the distal phalanges. Affected individuals usually have short stature and increased bone fragility, with fractures occurring after minimal trauma because of impaired bone remodeling and reduced bone elasticity [5].

The diagnosis is suggested by characteristic clinical and radiographic findings [6] and is confirmed by molecular analysis showing biallelic pathogenic or likely pathogenic variants in CTSK. When novel variants remain classified as variants of uncertain significance (VUS), integration of phenotype, segregation, population frequency, and computational evidence may support their role as strong candidate disease-associated variants, while functional confirmation remains necessary.

Here, we report a female child with clinical and radiological features consistent with pycnodysostosis. Genetic testing revealed two novel heterozygous CTSK variants in trans, establishing a compound heterozygous configuration.

## 2. Materials and Methods

Genomic DNA was extracted from peripheral blood leukocytes according to standard procedures. Clinical exome sequencing was performed using the ClinEx Pro capture kit (4bases, Manno, Switzerland) followed by high-throughput sequencing on an Illumina NextSeq 550Dx platform. Sequencing data were analyzed using the Geneyx Analysis CE-IVD software (rev. 7) pipeline. The sequencing performance showed a coverage of 94% of target regions at ≥50× and ensuring reliable detection of coding and splice-region variants. Sequence reads were aligned to the human reference genome and variants were annotated and prioritized according to allele frequency, predicted functional effect, inheritance model, and phenotype correlation. Variant filtering prioritized rare coding and splice-site variants compatible with an autosomal recessive skeletal dysplasia and with the patient’s phenotype. Candidate variants were evaluated using population databases, including gnomAD and ClinVar, and interpreted according to ACMG/AMP guidelines as implemented in Franklin by Genoox. In silico assessment included conservation analysis and multiple pathogenicity prediction tools. The two CTSK variants identified in the proband, c.85T>C (p.Trp29Arg) and c.679A>T (p.Ile227Phe), were confirmed by Sanger sequencing. Segregation analysis by Sanger sequencing demonstrated that the c.85T>C variant was inherited from the father and the c.679A>T variant from the mother, confirming the presence of the variants in compound heterozygosity, consistent with the autosomal recessive inheritance pattern of pycnodysostosis.

## 3. Case Report

The patient is the third child born to healthy, non-consanguineous parents of Nigerian origin. The parents could not recall the precise age at which the skeletal deformities first became apparent and were unaware of any history of bone fractures. Since the patient was first evaluated at an older age, reliable information on very early skeletal findings, including the timing of fontanelle closure or ossification, was not available from clinical history.

**Figure 1:**
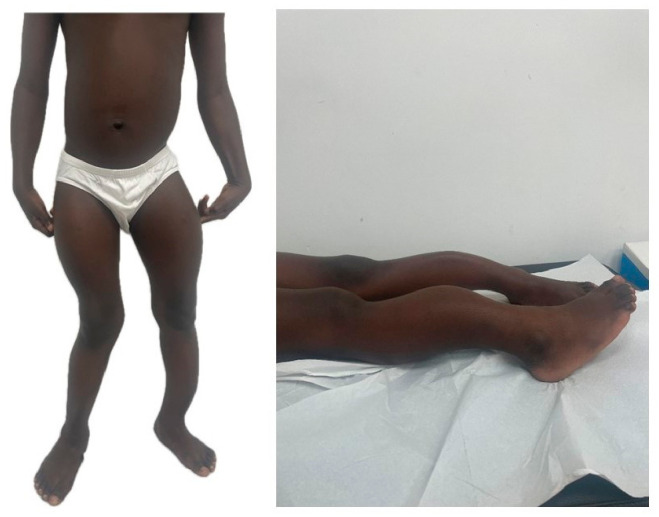
Limb deformities in a 12-year-old girl with clinical and radiological features consistent with pycnodysostosis. Both lower limbs are markedly bowed in the sagittal plane.

She was first evaluated in our clinic at 11.8 years of age. At presentation, she was in good general health. Her weight was 26.7 kg (5th percentile), and her height was 122 cm (<3rd percentile). She was prepubertal (Tanner stage P0, B0). Physical examination showed frontal bossing, brachydactyly, and prominent lower-limb deformities. Both legs were markedly bowed in the sagittal plane, which was the most evident abnormality (Figure 1, Table 1).

**Figure 2:**
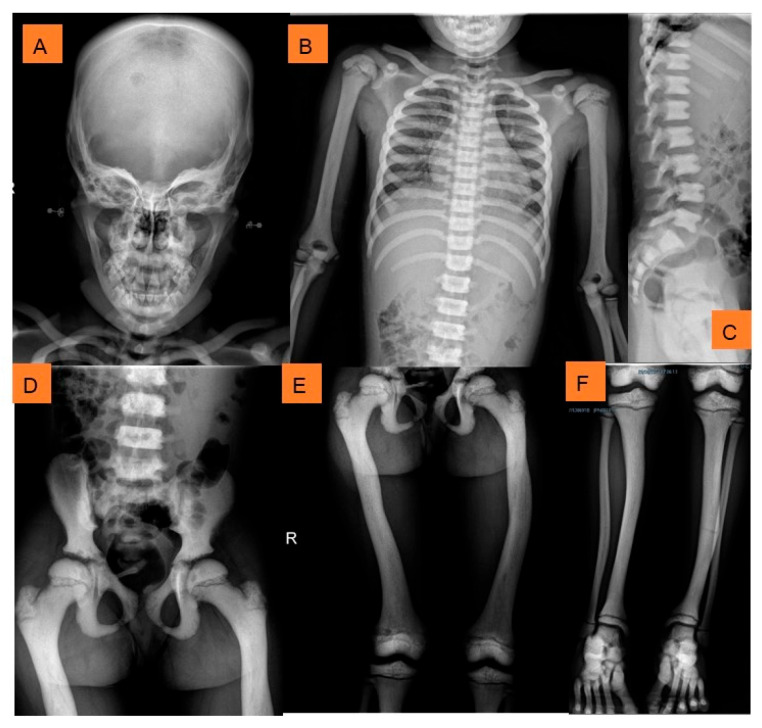
Radiographic features consistent with pycnodysostosis. (**A**) Posteroanterior skull view showing increased bone density and calvarial thickening. (**B**) Thoracic X-ray showing hypoplasia of the lateral (acromial) ends of the clavicles. (**C**) Lateral spine radiograph showing spool-shaped vertebral bodies with anterior fishtail deformities. (**D**,**E**) Radiographs of the pelvis and femurs showing diffuse osteosclerosis and a healed fracture with deformity of the left femur. (**F**) Tibia and fibula radiographs showing diffuse sclerosis, bowing, and a transverse midshaft fracture of the left tibia.

Radiological evaluation showed generalized osteosclerosis, most prominent in the long bones, which were bowed and showed cortical thickening with obliteration of the medullary cavity (Figure 2). The left femoral diaphysis showed a marked angular deformity, consistent with sequelae of a previous fracture and varus alignment. The vertebral bodies had a spool-shaped appearance. The ribs were thickened and sclerotic, and the clavicles were dysplastic with hypoplasia of their lateral ends.

Laboratory investigations showed the following values: WBC 6500/µL, neutrophils 2500/µL, hemoglobin 9.7 g/dL, MCV 79 fL, and platelets 216,000/µL. Bone metabolism markers were: calcium 7.07 mg/dL, alkaline phosphatase (ALP) 949 IU/L, magnesium 1.74 mg/dL, phosphate 5.38 mg/dL, albumin 45.8 g/L, parathyroid hormone (PTH) 234 pg/mL (normal range 12–88), and vitamin D 14.6 ng/mL (normal >30). Vitamin D supplementation was initiated because of deficiency.

Clinical exome sequencing identified two heterozygous variants in CTSK: c.85T>C (p.Trp29Arg) and c.679A>T (p.Ile227Phe). Both variants are currently classified as variants of uncertain significance (VUS) in ClinVar and according to ACMG/Franklin criteria [5,6].

Parental segregation analysis showed that c.85T>C was inherited from the father and c.679A>T from the mother, confirming a compound-heterozygous state in trans. This finding is consistent with the biallelic involvement expected in autosomal recessive pycnodysostosis, although the variants remain formally classified as VUS.

At the protein level, c.85T>C causes a missense substitution of tryptophan by arginine at codon 29 (p.Trp29Arg), located in the cathepsin K propeptide domain. The c.679A>T variant causes substitution of isoleucine by phenylalanine at codon 227 (p.Ile227Phe), located in the mature catalytic domain. The positions of these variants suggest possible disruption of both enzyme processing and catalytic function.

**Figure 3:**
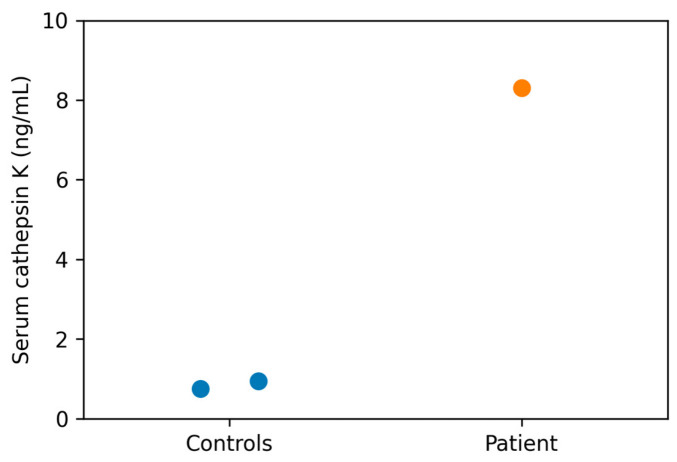
Exploratory measurement of serum cathepsin K. Serum from the patient and two age-matched controls was analyzed by ELISA. Cathepsin K concentrations were higher in the patient than in both controls. Given the exploratory design and the very small sample size, no inferential statistical test was applied.

To complement the in silico assessment of these variants, serum cathepsin K was measured in the patient and in two age-matched controls. Cathepsin K concentrations were higher in the patient than in both controls (Figure 3). However, this finding should be interpreted with caution, as it is based on a single affected individual and two controls. Therefore, the serum cathepsin K result represents a preliminary exploratory observation and should not be regarded as definitive functional validation of the CTSK variants.

## 4. Discussion

The typical age at diagnosis of pycnodysostosis is around 3–4 years; however, delayed recognition, as in the present case, is not uncommon [7]. Our patient had lived for several years separated from her parents in a country with limited medical resources. Social and economic difficulties likely contributed to delayed medical evaluation. Therefore, the age at presentation does not necessarily reflect the natural onset of the disease, and genotype–phenotype correlations should be made with caution.

Recent clinical series confirm that, although pycnodysostosis has a recognizable skeletal and craniofacial phenotype, disease severity and complications vary considerably, even among patients carrying the same CTSK variants. This observation supports the likelihood that additional genetic, endocrine, nutritional, or environmental modifiers contribute to the final phenotype [8].

The disease burden on this patient is substantial. Her physical abilities are severely impaired, and during the first months after diagnosis she frequently sought medical care for common respiratory infections and bone pain. Her quality of life is therefore markedly compromised compared with that of her peers.

The differential diagnosis includes skeletal dysplasias and metabolic bone diseases with overlapping features, including osteopetrosis, hypophosphatasia, osteogenesis imperfecta, selected craniofacial dysostoses, mucopolysaccharidoses, and vitamin D-dependent rickets. In the present case, the combination of short stature, generalized osteosclerosis, clavicular hypoplasia, long-bone deformities, and radiological evidence of fractures strongly supported pycnodysostosis. Radiographic imaging, laboratory evaluation, and genetic testing were essential for distinguishing among these conditions.

Review by an experienced pediatric radiologist was particularly useful in directing the diagnostic work-up toward appropriate molecular testing.

Recognizing pycnodysostosis is clinically important because, although the condition is generally considered non-progressive, fracture risk persists throughout life, and complications may modify the clinical course. Monitoring prepubertal linear growth is also important: recombinant human growth hormone (GH) therapy may improve growth velocity in selected patients, particularly when GH deficiency is present [7]. However, recent cohort data show heterogeneous responses to GH therapy, with limited benefit in some patients and occasional adverse effects; treatment decisions should therefore be individualized and accompanied by close endocrine follow-up [8].

The same recent series also reported pseudotumor cerebri in a substantial proportion of affected patients, suggesting that raised intracranial pressure may be an underrecognized complication of pycnodysostosis. Even in the absence of symptoms, periodic neurologic and ophthalmologic assessment may be prudent, particularly in patients with persistent cranial suture abnormalities or headaches [8].

Cathepsin K, encoded by CTSK, is synthesized as an inactive pre-proenzyme of 329 amino acids. The precursor contains a 15-amino acid signal peptide, a 99-amino acid propeptide, and a 215-amino acid mature catalytic domain. The signal peptide directs the nascent protein to the rough endoplasmic reticulum, where it undergoes post-translational processing. The propeptide region contains a conserved N-glycosylation site involved in lysosomal targeting through the Golgi apparatus. Enzyme activation in the lysosomal compartment requires cleavage of the propeptide between Arg114 and Ala115, releasing the mature enzyme. This maturation process is required for catalytic activity and for the physiological role of cathepsin K in extracellular matrix and collagen degradation [9].

Previous studies have identified CTSK variants distributed across the signal peptide, propeptide, and mature catalytic domains, including missense, nonsense, frameshift, splice-site, and stop-codon variants [8,10,11,12,13,14]. These reports highlight marked allelic heterogeneity, although recurrent or founder variants, such as c.244-29A>G, may predominate in geographically clustered populations [8,12].

The present case differs from founder-associated cohorts because the patient was born to non-consanguineous parents of Nigerian origin and carries two previously unreported missense CTSK variants in trans. This observation adds to the allelic heterogeneity of CTSK-related pycnodysostosis and supports the relevance of considering compound heterozygosity in non-consanguineous families with a compatible clinical and radiological phenotype.

The two variants reported here, p.Trp29Arg and p.Ile227Phe, affect the propeptide and catalytic domains, respectively. Trp29 lies within the propeptide domain, which is important for lysosomal targeting and enzyme maturation. Substitution of a hydrophobic tryptophan with a charged arginine may affect folding or zymogen activation. Ile227 lies in the mature catalytic domain, near regions involved in substrate interaction. Replacement of isoleucine by phenylalanine may introduce local steric effects and reduce structural stability, thereby potentially impairing enzymatic function.

The elevated serum cathepsin K observed in our patient is intriguing but should be considered exploratory. It may reflect altered expression, abnormal secretion, reduced clearance, or accumulation of dysfunctional protein. It cannot be taken as evidence of compensatory upregulation, and enzymatic activity assays will be required to distinguish among these possibilities.

Both c.85T>C (p.Trp29Arg) and c.679A>T (p.Ile227Phe) are currently classified as VUS in ClinVar and by ACMG/Franklin criteria. Available evidence supports their consideration as strong candidate disease-associated variants but is not sufficient to reclassify them as pathogenic or likely pathogenic.

The variants meet supporting criteria including PM2, because of extremely low allele frequency in gnomAD, PP3, because computational prediction tools support a deleterious effect, and PP2, because CTSK has a low rate of benign missense variation and missense variants are a known mechanism of disease.

VarSite analysis indicated that p.Trp29Arg affects a highly conserved residue (conservation score 1.0 from 172 aligned sequences), with no natural variants reported in gnomAD. The tryptophan-to-arginine substitution is highly unfavorable and has a disease-propensity score of 2.83, suggesting a high likelihood of functional impact. The p.Ile227Phe variant affects a conserved residue (conservation score 0.8 from 198 aligned sequences). Although the amino acid change is relatively conservative, the variant is extremely rare and has a disease-propensity score of 1.13, indicating possible functional relevance.

Taken together, the patient’s phenotype, radiographic findings, trans configuration of the two CTSK variants, rarity in population databases, conservation data, and computational predictions support p.Trp29Arg and p.Ile227Phe as strong candidate disease-associated CTSK variants. However, because both variants remain classified as VUS, these data should be regarded as supportive rather than definitive evidence of pathogenicity.

Functional validation remains necessary to confirm the predicted effects of these variants on cathepsin K expression, maturation, and enzymatic activity.

## Figures and Tables

**Table 1:** Phenotypic features of the patient compared with typical manifestations of pycnodysostosis.

Feature	Status in This Patient	Comment
Short stature	Present	Height < 3rd percentile at 11.8 years
Generalized osteosclerosis	Present	Diffuse radiographic osteosclerosis
Long-bone bowing/deformity	Present	Marked lower-limb bowing
Fractures	Present/radiological evidence	Healed left femoral fracture; transverse midshaft fracture of the left femur; transverse midshaft fracture of the left tibia
Clavicular changes	Present	Hypoplasia of the lateral/acromial ends
Cranial sutures/fontanelles	Not fully documented	Skull radiograph showed increased density and calvarial thickening; suture/fontanelle status not specified
Dental anomalies	Not documented	No dental abnormality reported in the available clinical record
Acro-osteolysis/brachydactyly	Partially documented	Brachydactyly present; acro-osteolysis not specifically documented radiographically
Craniofacial features	Present	Frontal bossing reported

## Data Availability

The raw data supporting the conclusions of this article will be made available by the authors on request.
